# Progress in germline stem cell transplantation in mammals and the potential usage

**DOI:** 10.1186/s12958-022-00930-5

**Published:** 2022-03-31

**Authors:** Wen Zhang, Ruotian Nie, Yihui Cai, Wenhai Xie, Kang Zou

**Affiliations:** 1 Germline Stem Cells and Microenvironment Lab, College of Animal Science and Technology, Nanjing Agricultural University, Nanjing, 210095 China; 2 College of Life Science, Nanjing Agricultural University, Nanjing, 210095 China; 3 School of Life Sciences, Shandong University of Technology, NO. 266 Xincun Road, Zibo, 255000 Shandong China

**Keywords:** Chemotherapy, Fertility, Homing efficiency, Livestock, Three-dimensional culture

## Abstract

Germline stem cells (GSCs) are germ cells with the capacities of self-renewal and differentiation into functional gametes, and are able to migrate to their niche and reconstitute the fertility of recipients after transplantation. Therefore, GSCs transplantation is a promising technique for fertility recovery in the clinic, protection of rare animals and livestock breeding. Though this novel technique faces tremendous challenges, numerous achievements have been made after several decades’ endeavor. This review summarizes the current knowledge of GSCs transplantation and its utilization in mammals, and discusses the application prospect in reproductive medicine and animal science.

## Background

GSCs initiate from primordial germ cells (PGCs) in embryonic stage. As a group of germ cells capable of self-renewal and differentiation into functional gametes, they are fundamental cells for reproduction.

Male germline stem cells, also called spermatogonial stem cells (SSCs), are the foundation of spermatogenesis in post-natal mammals. The main characteristic of SSCs is the ability to differentiate into spermatozoa while maintaining the capacity of self-renewal [1]. SSCs reside in a microenvironment consisting of Sertoli cells, Leydig cells as well as the basement membrane of seminiferous tubules [2]. The quantity and quality of SSCs are greatly affected by microenvironment. An optimal microenvironment is essential for maintaining the homeostasis of SSCs, and impaired function of microenvironment is usually accompanied with ageing. Since the 1950s, several methods have been used for identification of SSCs, including histological approaches, whole-mount analyses, and isotope pulse-chase monitoring, but none of the results was effective [1]. Until 1994, testicular transplantation was first explored as a method to identify SSCs through evaluating ability to re-establish spermatogenesis in recipient testes [3], and subsequently it was used as a tool for biomedical science [4], for example, for tracing the homing of donor SSCs and mechanism of spermatogenesis [5]. Methods for evaluation of SSCs homing efficiency in mice and the strategies for further improvement were reported, as well [6, 7]. Functions of different signaling pathways on the self-renewal or differentiation of SSCs can also be verified through this way [8, 9]. Furthermore, in order to detect subtle defects of SSCs in different culture systems, competitive transplantation can be applied [10]. Nowadays, livestock and some other animals, including non-human primates have been used as recipients for further research, and spermatogenesis could be observed in some species [11–13].

In 2004, a study indicated that mitotically active germ cells in postnatal ovaries were likely to be responsible for oocyte production [14]. However, the identity of putative germline stem cells in postnatal mammalian ovary was questioned by some researchers due to the lack of direct evidence [15–17]. In 2009, germ cells with capacity of self-renewal were isolated and purified from mouse ovaries, and their germline stem cell identity was proven using ovarian transplantation. Due to the fact that these mitotically active cells were capable of reconstituting oogenesis in the recipient’s ovaries, they were nominated as female germline stem cells (FGSCs) [18]. As a promising technique for fertility recovery and biological research, FGSCs transplantation is now feasible to obtain donor-derived offspring in mice [18, 19] and rats [20]. It is also a powerful tool to reveal the biological mechanism, for example, tracing the development and behavior of transplanted FGSCs in vivo enables to explore the trajectory of FGSCs homing [21].

Both SSCs transplantation and FGSCs transplantation have made some progress despite that a number of challenges still remain. Functional sperms can be produced in the recipient testes of many species, including dogs [12], tree shrews [22], and rhesus macaques [23]. Since these animals are good models for biomedical research, the success of transplantation on them is encouraging, especially in non-human primates. However, the success rate of obtaining donor derived offspring is actually very low [12, 13, 24]. On the other hand, oogenesis could be observed when human FGSCs were transplanted into human cortical tissue followed by xenografting the tissue into mice [25], while the application of FGSCs transplantation in the clinic remains undetermined. Therefore, further research is needed to raise the transplantation efficiency and ultimately lay a solid foundation for the application of GSCs transplantation in clinic. In this review, current progress achieved in GSC transplantation as well as its perspectives for future application in mammals are discussed.

## The process of germline stem cell transplantation

GSC transplantation can be simply described as below: it is a technique that aims to realize homing and germline transmission in vivo of donor SSCs or FGSCs. The quality of donor cells, the condition of recipients, and the transplantation technology are critical for transplantation success. Moreover, the assessment of colonization efficiency and functional analysis are necessary after transplantation.

### Preparation of donor cells

#### Isolation and culture of SSCs

SSCs are rare in the testis. The number of SSCs is only about 35,000 per testis, which only takes 0.03% among the whole cells of neonatal mouse testis [26]. An obvious increase in the number of functional SSCs occurs during postnatal development. However, the size of colonies that derived from transplanted SSCs appears to be independent of the age of the donors [27]. Considering the fact that only 10% of the SSCs remain after 7 days’ culture [28], various methods are applied to enrich SSCs in vitro for transplantation. It is noteworthy that in patients with cancer, sufficient healthy spermatogonia should be obtained from testicular tissue before chemotherapy [29].

Collection of SSCs usually starts from a two-step enzymatic digestion (collagenase and trypsin), which is followed by centrifugation and differential adhesion method to primarily enrich SSCs [3]. To purify SSCs, additional steps such as density gradient centrifugation, immunomagnetic bead-based sorting, or flow cytometry are usually applied [30–32]. The latter two methods rely on the surface markers such as ITGA6 [33], THY1 [34], MCAM [32], c-KIT [35], MHC-I [34], ITGAV [35], PLD6 [36], CD9 [37] to efficiently enrich SSCs for hundreds of folds [38].

In addition to cell purity, establishing an effective culture system is equally important for SSC proliferation. Usually two strategies are applied in traditional culture systems, adding growth factors necessary for self-renewal of SSCs in the culture medium [39, 40] and decreasing the influence of feeder cells or serum [41, 42]. Nowadays, many cell lines from SSCs have been established.

#### Isolation and culture of FGSCs

In 2009, a group of germ cells isolated from mouse ovaries with a diameter of 12-20 μm were characterized as mitosis active and can be cultured on fibroblast feeder layers. More importantly, they successfully recovered the reproductive ability of sterile recipient after transplantation into ovary, thus were named as FGSCs [18]. FGSCs are even more scarce than SSCs, only 50–100 MVH positive (MVH^+^) cells can be obtained from 6–8 adult mice while 200–300 MVH^+^ cells can be obtained from 9–12 neonatal mice, with FGSCs among them [18]. Subsequently, a more efficient sorting marker for the enrichment of FGSCs, Fragilis, was identified, and about 1100 FGSCs were collected from ovaries of 20 five-day mice [43]. These cells could be maintained on MEF feeder layers in 24-well plate for enrichment, and need to be purified to remove the feeders before transplantation.

The identity of FGSCs was identified using BrdU/DDX4 dual immunostaining. Subsequently, more markers were discovered, such as FRAGILIS, STELLA, OCT4 and SSEA-4 [44]. DDX4, FRAGILIS and OCT4 have been used in FACS- and MACS-based isolation methods. Although DDX proteins are usually localized in cytoplasm, studies revealed that DDX4 contains a transmembrane domain on the C-terminal, which could be recognized by the antibody for cell sorting [18, 25, 45, 46].

The culture system of FGSCs is similar to that of SSCs, which requires feeder cells as well as several growth factors for self-renewal [44]. Addition of LIF significantly increases the number of FGSCs colonies and GDNF is essential for FGSCs maintenance on MEF feeder layer [47]. Moreover, the use of BIO, a GSK3 inhibitor can increase the number of colonies as well [48].

### Preparation of recipients

Preparation of recipients is a critical step towards successful transplantation as the microenvironment remarkably affects colonization efficiency. Different strategies have been developed to obtain a microenvironment-intact recipient that lack of endogenous spermatogenesis, including busulfan treatment [49], testicular irradiation [50], gene editing [51], and heat shock treatment [52]. In some instances, CdCl_2_ may be used in combination with busulfan for sterile models with eliminated Sertoli cells [53]. These techniques have both advantages and disadvantages. As a chemotherapeutic drug, busulfan is commonly used for eliminating endogenous spermatogenesis. It is worth to mention that the optimal dose of busulfan remains to be determined in large animals because of its toxicity and the inevitable recovery of endogenous spermatogenesis [52, 54]. Moreover, at least 35 days are required before the treated animals can be used as recipients according to the epithelial cycle of spermatogenesis. Compared with that in busulfan treatment, spermatogenesis recovery is inevitable in heat shock treatment, but it is safer and shortens the time required for recipient preparation [52]. Athough local irradiation is more efficient and safer, it is the most expensive of the four methods since it needs special equipment [50]. Knockout of pivotal genes has also been widely used for sterile recipients, as well. Both W mutant mice [51] and *Nanos2* knockout male animals [55] are infertile with intact testicular microenvironment, and have been used as efficient recipients for SSCs transplantation. For preparation of female recipient mice, intraperitoneal injection of busulfan (30 mg/kg) is accompanied with cyclophosphamide (120 mg/kg) to eliminate endogenous germ cells in ovary [18, 21], since cyclophosphamide can directly induce oocytes in primordial follicles death, avoiding endogenous oogenesis to some degree [56].

### Transplantation technique in rodents

For SSCs transplantation, selection of the appropriate injection site in recipient testis greatly influences the transplantation efficiency. Considering the differential reproductive anatomy of species, injection sites are optional, including seminiferous tubules, efferent ducts and rete [57]. Seminiferous tubules are widely chosen in mice since they spread all over the testis and are most accessible for investigators. However, the disadvantages are time-consuming and requiring extra liquid for injection with a number of incisions to ensure the tubules are filled as much as possible. The efferent ducts are considered as more efficient injection sites but careful dissection is demanded. Excessive internal testicular pressure may cause ischemia and damages microenvironment or harms donor cells, that is why it may be necessary to inject with the aid of a pressure injector. The last one is rete, where allows quick filling of tubules. Notably, this injection site is only applicable in species with the rete closer to the surface of the testis, such as mice and rats [58].

According to our experiences, the efferent ducts of a mouse recipient appear to be the most convenient site, because it requires less cells to achieve high filling efficiency, and could be finished with appropriate needles under stereoscope.

### The assessment of colonization efficiency

Once GSCs are transplanted into the tubules successfully, their abilities of homing, proliferation, gametogenesis and producing offspring need to be verified. The efficiency of transplantation is assessed mainly by the colonization of donor GSCs and the production of spermatozoa or oocytes. The recovery of gametogenesis in recipient testes or ovaries is usually assessed with histological analysis [18], and it is also important to test whether those spermatozoa or oocyte are donor-derived. Usually, donor cells carrying fluorescence or containing a LacZ transgene are used for tracking donor-derived cells [25, 55, 59]. And the success of germline transmission needs to be confirmed through detection of the reporter gene in offspring at DNA level, using PCR or Southern blot [18]. Although PCR is fast, convenient and economical, the accuracy of is not satisfying, while Southern blot is the gold standard to confirm the inserted DNA sequences.

## Current progresses of germline stem cell transplantation in domestic animals

GSCs transplantation is well studied in rodents, whereas some obstacles still need to be solved in livestock and poultry. Here we highlight the progress of GSCs transplantation in animal husbandry.

Livestock biotechnology has greatly progressed and SSCs manipulation is one of the most promising techniques because it can be used as the basis for transgenic technology in the future [60]. However, the long-term cultivation of livestock SSCs in vitro is still a challenge in many species. The culture system established originally in rodents is impossible to transfer directly to livestock, except for growth factors, many other factors are probably needed to be modified [61, 62].For example, a stable long-term culture system for porcine SSCs was eventually established, through searching for necessary cytokines, level of serum, culture temperature as well as optimal feeder cells [63]. Neonatal Sertoli cells were proven to be a type of efficient feeder cells for porcine SSCs, and the culture medium supplemented with KSR and a combination of GDNF, GFRα1, bFGF, and IGF1 was used. Porcine SSCs formed consistent colonies showing typical grape-like morphology in vitro for over 2 months under this culture condition [63]. This encouraging result represents huge progress of long-term culture systems of SSCs from large animals.

In addition, preparation of recipient animals is another obstacle since many methods applied in rodents are inappropriate. Busulfan treatment [64] and testicular irradiation [65] are not ideal for large animals because the structure of their seminiferous tubules may be destructed. A recent study on the effect of busulfan on the depletion of pig endogenous SSCs claimed that 3 mg/kg of busulfan can be injected through intratubular to establish recipient pigs, which is a safe dosage. However, no donor-derived sperm was found even though donor-derived SSCs colonized in the recipient testes for over 4 months. The author postulated that recovery of endogenous germ cells possibly happened [66]. This case supports the view again that neither busulfan treatment nor testicular irradiation could prevent the recovery of endogenous SSCs [54].

However, SSCs transplantation has succeeded in boar [67], ram [11], and bull [55]. It is worth mentioning that seminiferous tubules of some species locate deeper in the testis or with smaller tubular diameter. Rete is the optimal injection site with the aid of ultrasonographic-guided technique when it is difficult to inject directly into the seminiferous tubules [68].

Until now, the function and vitality of donor SSCs have been verified in some species of livestock. The production of embryos or even progeny has been reported in rams [11] and boars [67]. If this technique is well applied in dairy cattle, producing offspring with superior traits will be remarkably accelerated. It is also applicable in beef cattle, swine, sheep, and goats since it not only shortens the time of production, but also efficiently extends the use of donor-derived sperm worldwide [54].

## Technical improvement

Both GSCs and supporting cells contribute to spermatogenesis in recipient testes. Thus, a lot of methods have been explored to improve their functional activities. The quality of donor cells as well as the microenvironment in the recipient testes are critical for success. Besides, the efficiency of colonization may be greatly impacted by the levels of hormones and timing for transplantation.

### Improvement in the quality of donor cells

Donor cells may lose their characters of stem cells during the transplantation process while only part of them persist and successfully regenerate. To solve this problem, additional treatments are required to increase the proportion of undifferentiated stem cells before colonization. Recently, a study reported that the transient suppression of differentiation using WIN 18,446, a chemical inhibitor of retinoic acid synthesis was able to expand the GFRa1^+^ SSC pool and increase the repopulation efficiency [7], suggesting that SSCs tend to differentiate before they arrive in niche.

Apart from suppression of stem cell differentiation, improvement of ability to self-renew is another option. Our previous study identified that activation of AKT3 could promote FGSCs self-renewal [47], which indicates that addition of SC-79, an AKT3 specific activator, might improve transplantation efficiency of FGSCs.

Establishment of a culture system that mimics the microenvironment in vivo may be another method for more active donor cells. Cell morphology, proliferation activity, differentiation ability, gene expression, stress response and other factors are potentially affected when cells are maintained on conventional culture system. Numerous solutions are proposed, including the use of different conditioned media [41, 42], feeder cells [69], and growth factors [39], but the process of spermatogenesis has not been accomplished in two-dimensional culture system [70, 71].

Recently, three-dimensional (3D) culture was applied to provide an ideally spatial environment for both testicular organoid and bioengineered ovaries [72, 73]. Many biological processes can be more accurately studied through the use of 3D culture system such as the spermatogenic process [74]. A novel 3D culture system is based on a soft agar culture system (SACS) [75], with two major constituents of SSCs niche, somatic cells [72] and the extracellular matrix (ECM) [76]. SACS makes it more efficient than some other 3D culture systems that are based on collagen, gelatin or matrigel, attributing to the thick layer for germ cells and supporting cells to embed in [77]. To extract ECM components, a novel three-dimensional multilayer model, called decellularized testicular matrix (DTM) scaffold, was used. This model well preserves the 3D structure as well as biocompatibility. Using this technology, SSCs of mice and many other species such as porcine [78], chickens [79], primates [77], and even human [80] can be cultivated in this system.

Similar methods can be applied to the 3D culture of ovarian organoids. As the prerequisite step, decellularization method only succeeded in ovarian pieces and sometimes cortical slides [81]. In order to acquire a whole decellularized ovary, several methods were explored, and ultimately physical and chemical methods were combined to remove cellular components [73, 81]. In contrast to 2D culture system, using 3D culture system greatly promotes the cell expansion and enriches sufficient cells for transplantation. Moreover, it eliminates potential variability and contamination of feeder cells [82]. Therefore, GSCs cultured in a 3D system may be better resource for SSCs transplantation.

Differentiation of FGSCs into oocyte has been achieved in 2D system into germinal vesicle (GV)‐stage oocytes [83], indicating the potential to obtain functional oocytes in vitro using a 3D system in the future. FGSCs transplantation may benefit even more from 3D culture in the future. For instance, donor cells cultured in a 2D system need to be resuspended with buffer solution after digestion from the feeder cells. This makes it hard to ensure sufficient FGSCs are maintained in the ovarian cortex. To solve this problem, Matrigel Matrix is an ideal material for stem cell culture [84] and maybe FGSC transplantation as well, because it can convert from a liquid state to a solid state as the temperature increases from 4℃ to 37℃. And it is easier to confirm transplanted FGSCs are localized in the injection site.

### Restoration of damaged microenvironment

An intact microenvironment is essential for homing and survival of GSCs, but some recipients may suffer from supporting cell defects [85]. Therefore, restoring the impaired niche is an effective way to improve the outcome of GSCs transplantation. Sertoli cell is known as the most important component of testicular niche. A previous study revealed that transplantation of testes cells derived from perinatal mice efficiently repaired the microenvironment of the recipient and generated donor-derived spermatogenesis [86], suggesting that co-transplantation of Sertoli cells may contribute to a higher success rate of GSCs transplantation. Besides, co-transplantation of mesenchymal stem cells (MSCs), also improves SSCs transplantation efficiency in mice. Moreover, even higher efficiency can be achieved when MSCs are treated with TGFß1, which makes them lose their migratory property and retain in the testis [53, 87].

For female, the application of MSCs is also an important method that effectively improves ovarian function due to their abilities of differentiation and restoring endometrial function [88]. Besides, human endometrial MSCs can differentiate into granulosa cells and improve the renewal of GSCs [89]. These findings imply the possibility of co-transplantation of MSCs in females as well.

Although co-transplantation facilitates in the restoration of fertility, it is required to thoroughly check effectiveness and safety before clinic use, especially on genetic and epigenetic levels.

### Regulation of hormones

The proliferation and differentiation of GSCs are related to the regulation of various hormones such as testosterone and follicle-stimulating hormone (FSH). Researchers revealed that the efficiency of colonization is notably enhanced when the recipient mice were treated with leuprolide, a gonadotropin-releasing hormone (GnRH) agonist [90]. It was also verified in irradiated rats [91] and monkeys [92], implying the potential of this method in restoring fertility of patients after cancer treatment [93]. However, there are still some issues in monkeys assays because the authors proposed that the proportion of filled tubules might have a greater influence on the colonization efficiency [24, 94]. In addition to leuprolide, FSH appears to be useful in facilitating the process of spermatogenesis in infertile mice [95].

Hormone can be used to regulate the integrity of the niche. During the process of SSCs homing, blood-testis barrier (BTB) hampers the transplanted cells to migrate to the niche in the seminiferous tubules [96], and researchers found that using acyline, another GnRH agonist that can transiently damage BTB by modulating the expression of claudin proteins, and improved the efficiency of SSCs homing [97].

Despite that the above-mentioned hormones may facilitate transplanted SSCs to colonize in the niche, the effects of hormones in infertile mice could be affected by different factors. For example, our previous study revealed that androgen level in rodent testis affected Sertoli cells on the expression level of ITGB1 [98], which is a pivotal surface protein for SSCs homing via regulating cellular interaction [99]. Therefore, maintenance of appropriate hormone level in recipients, especially patients after chemotherapy, is probably a prerequisite for successful transplantation of SSCs.

Although much remains unknown about the microenvironment of FGSCs, our previous study demonstrated that Cadherin-22 (CDH22) is a potential transmembrane protein in FGSCs niche [100]. Similarly, the expression level of surface marker on FGSCs is important for maintenance and homing of transplanted FGSCs.

### Selection of the appropriate timing of transplantation

With the purpose of increasing the efficiency of colonization and spermatogenesis, the appropriate time gap between the use of chemotherapeutic drugs and transplantation time was critical. A delay in the SSC transplantation timing was found to decrease the receptivity of recipient testes [101]. Similar observations were found in FGSCs transplantation [102].

## Conclusion

**Fig. 1:**
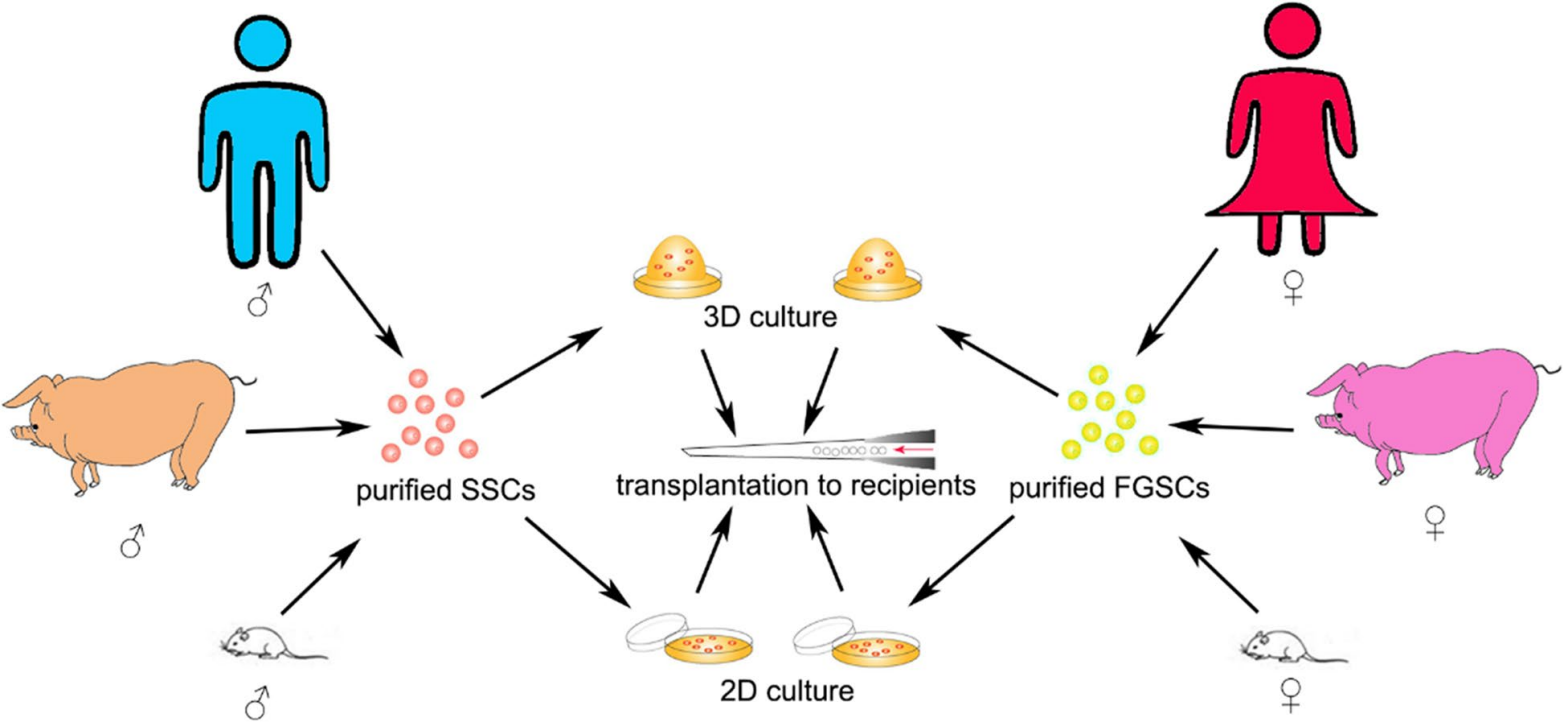
An illustration of GSCs purification, culture and transplantation is summarized. GSCs transplantation is a powerful tool for human fertility cure, animal breeding, and laboratory research. Isolation, purification, in vivo maintenance, and cryopreservation of GSCs are the prerequisites of transplantation.

There are still many unaddressed questions regarding GSC transplantation, especially the efficiency and safety. The efficiency of colonization is affected by various factors, including the microenvironment, endogenous GSCs, viability of donor GSCs and so on. Meanwhile, safety of this technique need full evaluation especially when it is used for assisted reproductive technology [103]. In our previous experiments, hair loss was observed in some recipients several weeks after transplantation. We speculated that it might be associated with immunological rejection. Currently there are still many difficulties in GSC transplantation, but it is a prospective technology for clinic and animal science in the future. If combined with gene editing and other assisted reproductive technologies such as in vitro fertilization and cryopreservation, GSC transplantation can not only benefit clinical medicine, for example patients who suffer from reproductive diseases or received chemotherapy will hopefully be cured through this way, but also improve livestock industry. An illustration of GSCs purification, culture and transplantation is summarized (Fig. 1).

## Data Availability

This review was based on published data.

## Abbreviations

GSCs: Germline stem cells
PGCs: Primordial germ cells
SSCs: Spermatogonial stem cells
FGSCs: Female germline stem cells
AI: Artificial Insemination
SACS: Soft agar culture system
ECM: Extracellular matrix
DTM: Decellularized testicular matrix
GV: Germinal vesicle
MSCs: mesenchymal stem cells
GnRH: gonadotropin-releasing hormone
FSH: follicle-stimulating hormone
BTB: blood-testis barrier
